# Pharmacotherapy in Stress Urinary Incontinence; A Literature Review

**DOI:** 10.1007/s11934-024-01205-9

**Published:** 2024-05-10

**Authors:** Seyed Sajjad Tabei, Wesley Baas, Ayman Mahdy

**Affiliations:** 1 Division of Urology, Department of Surgery, University of Cincinnati College of Medicine, 231 Albert Sabin Way, Cincinnati, OH 45267 USA; 2 R. Bruce and Barbara Bracken Endowed Chair in Surgical Urology, Department of Surgery, University of Cincinnati College of Medicine, 231 Albert Sabin Way, ML 0589, Cincinnati, OH 45267 USA

**Keywords:** Stress urinary incontinence, Urinary incontinence, stress, Urinary incontinence, Pharmacotherapy, Duloxetine, Drug therapy

## Abstract

**Purpose of Review:**

Stress urinary incontinence (SUI) is a commonly observed condition in females, as well as in males who have undergone prostatectomy. Despite the significant progress made in surgical techniques, pharmacotherapy has not yielded substantial outcomes within the clinical domain. This review aims to present a comprehensive overview of the existing pharmacotherapy options for stress urinary incontinence (SUI) and the emerging therapeutic targets in this field.

**Recent Findings:**

One meta-analysis demonstrated that α-adrenergic medications are more efficacious in improving rather than curing SUI symptoms. One trial showed reduced pad weight gain with PSD-503, a locally administered α-adrenergic receptor agonist. New data show that duloxetine’s risk outweighs its benefits. One small-scale trial was found to support the use of locally administered estriol in improving subjective outcomes. Emerging targets include serotonin 5HT_2C_ agonists, selective inhibitors of norepinephrine uptake, and myostatin inhibitors.

**Summary:**

Only one of the evaluated drugs, duloxetine, has been approved by some countries. Currently, trials are evaluating novel targets. Systemic adverse effects such as gastrointestinal upset with duloxetine and orthostatic hypotension with ***α***-adrenoceptor agonists have hampered the efficacy of drugs used to treat SUI in women and men.

## Introduction

Stress urinary incontinence (SUI) is a debilitating urological disorder defined as the involuntary leakage of urine upon activities that may increase the intra-abdominal pressure, such as exertion, sneezing, or coughing [1]. Various anatomical and physiological factors result in this condition, but the common endpoint of all etiologies is the inability of normal urinary control mechanisms to overcome the previously tolerated urinary pressure load. In these cases, there is often an interplay of various factors, which ultimately present as SUI. Non-modifiable determinants such as female sex and Caucasian race and modifiable factors such as smoking, obesity, and chronic constipation have all been shown to influence the progression to clinical presentation. Importantly, pregnancy, normal vaginal delivery, and past history of pelvic procedures such as hysterectomy have been shown to promote SUI in women [2]. Also, pelvic organ prolapse (POP), which can occur commonly after menopause or delivery, highly correlates with the concurrent presence of SUI, and in some cases, POP repair results in the unmasking of the urethral deficit and subsequent presentation with SUI [3]. A 12-year survey between 2005 and 2016 including more than 15,000 women in the US revealed that more than half of the respondents had experienced a form of incontinence. Moreover, around 1 in 4 respondents reported having a stress-only UI [4]. SUI significantly affects patients both in psychosocial and financial terms. Data evaluation in 2011 indicated that SUI management expenditures in the US were in excess of $12 billion per year [5].

Men, on the other hand, are prone to developing SUI often due to iatrogenic causes such as prostatectomy [6]. 

Although surgical methodology has progressed and conservative measures like pelvic exercises have been developed to treat SUI, the evidence-based clinical applicability of pharmacotherapy for SUI appears to be limited. This review looks to explore the current pharmacological arsenal in treating SUI and emerging experimental targets.

## Literature Description

.

### α-Adrenoceptor Agonists (α-AR Agonists)

Human studies have shown that three different subtypes of α-AR are expressed on the urethra, with α_1A_-AR being the most common in both sexes. There have been attempts to address SUI by targeting these receptors. However, the abundance of these receptors in other organs has hindered its efficacy as an established therapeutic option for treating SUI [7, 8]. Animal models had previously shown smooth muscle contractile changes in the urethra after stimulation of these receptors by noradrenaline (NA) [9].

In a 2011 study by Robinson et al., an experimental drug named PSD503 (phenylephrine 20% weight/weight) was proposed as a vaginal topical gel for treating SUI in women. In the phase II clinical trial, a total of 14 women were evaluated over a span of 20 months. Data indicated that treatment with this compound resulted in reduced pad weight gain compared to placebo (median weight reduction %: PSD503- 54.33%, placebo- 38%) [10]. Despite favorable outcomes, poor patient recruitment and follow-up were considered limiting factors in this study [11].

**Fig. 1:**
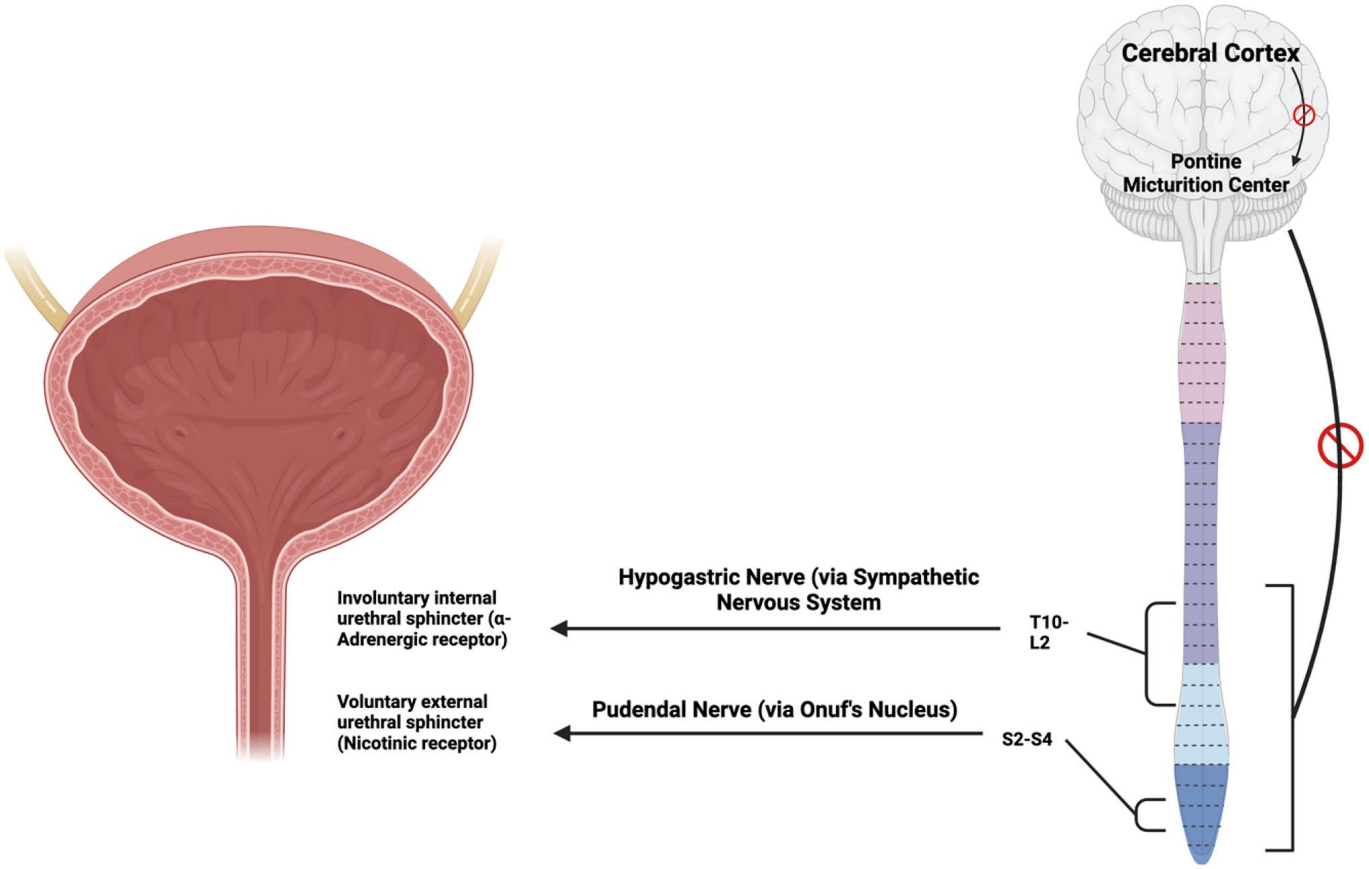
Voluntary and involuntary control of the urethra. The inhibitory role of the central control mechanisms has been outlined—illustration created in Biorender.com

By implementing new data synthesis techniques, a network meta-analysis of pharmacologic and non-pharmacologic UI treatments was conducted in 2019. The analysis was stratified based on symptom resolution or improvement, and the quality of the outcomes was reported as “standard of evidence” (SoE) in accordance with the Agency for Healthcare Research and Quality’s recommendations. Two separate studies encompassing a combined sample size of 736 patients were examined to assess and compare the rates of “cure” among patients with stress urinary incontinence (SUI) who received treatment with α-agonists vs. those who did not receive any intervention. The results revealed a moderate SoE in favor of α-agonists when they were used to achieve “cure” in these patients (OR:1.22; 95% CI: 0.47–3.03). The results were more convincing when the efficacy was evaluated for symptomatic “improvement” in SUI patients. The improvement data analysis comprised seven studies with 5035 cases (OR:2.28; 95% CI: 1.60–3.30). This cure/treatment analysis revealed that α-agonists may be useful if symptom relief rather than complete resolution is the goal of treatment [12] Fig. 1.

### β-Adrenoceptor Agonists and Antagonists

From a pharmacological standpoint, β-AR antagonists can potentially offset the relaxation effects of the endogenous NA stimulation and further enhance the α-AR urethral response of contracting the internal urethral sphincter. Drugs such as sotalol and propranolol have been studied in the past but have shown no meaningful response in the treatment of SUI [8]. The 5th edition of Walters & Karram Urogynecology and Reconstructive Pelvic Surgery textbook has outlined propranolol 10 mg b.i.d. to 40 mg t.i.d. daily as dosages that may help improve SUI symptoms [13]. However, no trials over the last 5 years have confirmed such a statement.

On the other hand, β-AR agonists such as clenbuterol have also been studied in the context of SUI treatment. Rabbit models suggested that selective β_2_-AR agonism, such as clenbuterol, propagated the external urethral contractile force [14]. There has been a lack of new human studies on this drug class over the recent years.

### Antidepressants

An increase in norepinephrine levels can potentially ameliorate the contractile response through central nervous system mechanisms in the Onuf’s nucleus [15]. Serotonin-norepinephrine reuptake inhibitors (SNRIs) and tricyclic antidepressants (TCAs) are two drug classes that have been extensively researched due to their shared properties [8, 16]. Kornholt et al. published a study in 2019 looking into the changes in urethral opening pressure caused by imipramine (TCA). The effects of a single dose of imipramine 50 mg and placebo on 16 healthy women with BMIs in the normal range were investigated using a randomized, double-blind, placebo-controlled cross-over design. A 6.5 cmH2O rise in urethral pressure at rest and a 7.9 cmH2O increase in the squeeze condition were seen compared to the placebo. The observed increase in urethral opening pressure did not reach statistical significance (p-value: 0.07 at rest, p-value: 0.06 under squeeze condition). Despite the limited sample size, the researchers reached the conclusion that the administration of imipramine is not recommended for cases of stress urinary incontinence (SUI) [17].

Duloxetine (SNRI) has also been extensively studied in the setting of off-label use for SUI and has been approved by the European Union. Human studies have shown using pressure reflectometry methods that duloxetine is superior to midodrine and placebo in increasing urethral tone [18]. The physiological pathway through which duloxetine exerts its effect may be through maintaining NE and 5HT levels in the CNS and Onuf’s nucleus. In addition, recent evidence suggests that maintaining serotonin levels plays a role in increasing urethral opening pressure via the 5HT2C pathway, which could explain the success of duloxetine and SNRIs in treating SUI [19]. In a Cochrane review on SNRIs and their role in SUI that was last revised in 2008, Mariappan et al. concluded that duloxetine should be considered as an effective means of treatment [20]. However, there has not been any update in this regard over the last 5 years.

Duloxetine is one of the few drugs that has been studied in the context of post-prostatectomy SUI. Research was undertaken involving a cohort of males who had undergone prostatectomy. The study sample consisted of 94 patients who were subjected to a treatment regimen involving the administration of duloxetine at a dosage of 30 mg once weekly immediately following the surgical procedure, followed by subsequent dosages of 60 mg once weekly. Review after a month of treatment showed that patient satisfaction was achieved in a third of patients, but the remainder discontinued the drug due to dissatisfaction or side effects. Around 15% of patients had experienced nausea, fatigue, light-headedness or dry mouth [21].

Despite clinical efficacy, side effects with duloxetine appear to be a concerning issue. A review of 6395 SUI patients treated with duloxetine revealed that adverse events occurred in 22.1% of patients and were resolved upon cessation of treatment. Common events included nausea, vomiting, constipation, and dry mouth [22].

#### Hormones and Hormone Modulators

In a prospective observational study by West et al. in 2023, the effects of a 12-week course of vaginal estriol cream 0.5 mg on SUI in postmenopausal females were measured. Forty-six postmenopausal women with pure SUI or stress-predominant mixed urine incontinence (MUI) who were not receiving additional incontinence therapy were included in the study. The primary endpoint was evaluating the vaginal pH levels and changes in the “Urogenital Distress Inventory-6 (UDI-6) stress domain scores”. The findings demonstrated significant improvement UDI-6 (dropping from a median of 83.3 to 33.3, *p* ≤ 0.001). Furthermore, vaginal pH levels notably reduced (from a median of 5.1 to 4.9, *p* ≤ 0.001) following the 12-week treatment. However, there was no mention of how the pH and urinary symptoms correlated with each other following estrogen therapy [23].

Enobosarm, alternatively referred to as Ostarine or MK-2866, is an experimental pharmaceutical compound categorized as a selective androgen receptor modulator. Its primary purpose is to activate androgen receptors in muscle and bone tissues. In a Phase 2 clinical study, researchers explored enobosarm for treating SUI in postmenopausal females. For 12 weeks, postmenopausal women with SUI received 3 mg of enobosarm orally. Stress incontinence episodes per day were measured using a 3-day voiding diary. All 17 subjects completing the 12-week treatment reported at least a 50% decrease in daily stress leaks. The average number of stress leaks experienced per day fell by 83% after 12 weeks, with a mean baseline of 5.08 leaks per day reducing to 0.88 leaks per day. This improvement was durable during the 40-week follow-up. Some patients even achieved a negative bladder stress test (BST) at 12 weeks, signifying significant improvement. Quality of life also improved, evident from the QOL instruments. The median female sexual function index (FSFI) score, measuring sexual function, notably increased. The MESA stress score, assessing stress and anxiety, significantly decreased. The administered treatment demonstrated a high level of tolerability, as no significant side events were seen, affirming its safety profile [24]. However, it was later reported in 2018 that the “ASTRID (Assessing Enobosarm for Stress Urinary Incontinence Disorder)” Trial was called off by its sponsor due to unconvincing results [25].

Testosterone possesses the capacity to elicit anabolic effects on the musculature of the pelvic floor. Kim et al. analyzed information from the 2012 “NHANES (National Health and Nutrition Examination Survey)” dataset, encompassing more than 2,300 women. The results suggested that women with lower levels of serum testosterone had a greater likelihood of experiencing stress and mixed urinary incontinence [26]. A 2018 clinical investigation (ClinicalTrials.gov Identifier: NCT03116087) examined testosterone therapy in postmenopausal women with SUI and low testosterone [27]. However, there have been no results published yet.

In a non-randomized clinical trial, Gažová and colleagues investigated the potential of CEL complex, an herbal compound, in managing SUI among female participants. The study findings demonstrated significant reductions in incidences of stress urinary incontinence (SUI) and improved alleviation of symptoms. These effects were attributed to the proposed mechanism of action of the herbal ingredient, which is believed to augment natural testosterone levels and influence the sphincteric muscles [28].

#### Experimental Targets; TAS-303, Muscle Growth Promoters & 5HT_2C_ Agonists

One of the recent developments in experimental therapeutics for SUI includes the development of TAS-303. This drug is a selective inhibitor of norepinephrine (NE) uptake with minimal central nervous system activity. In 2018, Mizutani et al. reported that in-vitro studies of this drug on murine models showed promising results by increasing the urethral closing pressure and leak point pressures in vaginal distension (VD) rats with superior efficacy than duloxetine. The VD rats were used to simulate the dysfunctional urethra following vaginal delivery. To assess the cardiovascular side effects, heart rate and blood pressure were monitored in dogs treated with TAS-303 which showed no statistically significant change [29].

Following the success in animal models, Yono et al. published the phase I clinical trial results of TAS-303 in 2020. A total of 16 women (treatment n: 8; placebo n: 8 patients) were enrolled in this randomized, double-blinded, cross-over trial. Each patient received 18 mg TAS-303 orally and was evaluated for MUCP after 6 h. There appeared to be an increase in the MUCP in the treatment group (3.473 ± 12.154 cmH2O) and the placebo group ( 2.615 ± 9.794 cmH2O ). However, the comparison of both groups was insignificant (p-value = 0.80). Additional analysis also demonstrated that there was no difference regarding drug contractile effect between the proximal, middle, and distal urethra [30].

The preliminary findings from the phase II clinical trial of TAS-303 were given in 2022 by Takahashi et al. (ClinicalTrials.gov Identifier: NCT04512053). The objective of this study was to evaluate the effectiveness of TAS-303 at a dosage of 18 mg once daily over 12 weeks. The medication group comprised 110 female participants, while the trial’s placebo arm included 111 female participants. Japanese women older than 20 and positive 1-hour pad tests over the prior three months were deemed eligible to enter this study. Moreover, if the individuals met the mentioned criteria and presented with a mean stress urinary incontinency episode frequency (SUIEF) per 24 h of ≥ 1 in the weekly bladder diary, they were considered suitable to enter the treatment phase. Following a 12-week period, it was observed that the medication group experienced a decrease of 57.50% in the average daily SUIEF, whereas the placebo group displayed a reduction of 47.35%. Statistical analysis of the collected data revealed a significant difference between the two groups (p-value = 0.047). The presentation did not provide specific details regarding the side effects; however, it was indicated that both groups experienced them at comparable rates. Compared to the placebo arm, the TAS-303 group showed greater improvement in subjective criteria [31].

Efforts have been recently made to evaluate experimental myostatin inhibitors for treating SUI in murine models. One example is the activin type II receptor blocker Bimagrumab, which promotes muscle growth by blocking the myostatin pathway [32]. Yang et al. conducted a study wherein rats were administered bimagrumab, and the resulting outcomes were compared to those observed with other muscle growth promoters, namely clenbuterol and a derivative of 5-hydroxybenzothiazolone (5-HOB). After two weeks, those treated with bimagrumab showed a higher leak point pressure than rats that had undergone treatment with clenbuterol or 5-HOB. This success was also confirmed when the tissues were histologically evaluated, which were in favor of muscle repair. However, significant overall weight gain was a limiting factor in this study [33].

Another experimental class of drugs that have recently been studied are the 5-HT_2C_ receptor agonists. This class of drugs has shown the potential to increase the leak pressure point in vitro and in animal models. A study in 2018 showed that vaginally distended rats treated with Lorcaserin (Belviq^®^), a 5-HT_2c_ agonist which at that time was FDA approved for treating obesity, showed significantly higher leak point pressures than controls. They concluded that this drug promoted urinary control via its effect on the pelvic muscles and external urethral sphincter control [34]. The FDA withdrew its approval of Lorcaserin in 2020 after studies showed significant cancer occurrence with this drug and disproportionate risks to benefits [35]. Another study also confirmed the effect on the urethra by treating animal models with ASP-2205 fumarate, a selective 5-HT_2C_ agonist [36]. TAK-233, also known as OP-233, has a much higher selectivity for human 5-HT_2c_ receptors and has shown improvement in animal urethral closure, suggesting applicability in cases of SUI. The phase I trial (Clinical trials ID: NCT02113020) was undertaken in 2014; however, there are no published results in this regard. It was announced in 2016 that Takeda and Frazier had partnered to develop this breakthrough drug [37, 38].

## Discussion

The accepted management options for SUI are dictated by the current understanding of the pathophysiology of this condition. Similarly, the bulk of research contribution over the last five years has been focused on surgical correction of this disorder rather than exploring pharmacotherapy options for SUI. However, the culmination of years of research on pharmacotherapy options has had a minimal impact in the clinical setting. The revised edition of the guideline on the management of female stress urinary incontinence (SUI) issued by the American Urological Association (AUA) in 2023 does not include any reference to medication as a conservative treatment option [39]. However, the European Association of Urology is more detailed on non-surgical options for women with SUI [40]. Their stance regarding hormonal options echoes the findings from a Cochrane review published by Cody et al. in 2012. It was concluded in this study that systemic estrogen therapy or combined estrogen/progesterone may worsen incontinence outcomes. On the other hand, limited use of locally delivered estrogen proved beneficial for overall incontinence outcomes (RR 0.74, 95% CI 0.64 to 0.86) [41]. The European guidelines also note that duloxetine may be used as a form of treatment for SUI. This is in contrast to the US, where duloxetine’s usage in these circumstances has not been approved by the FDA [42].

Multiple reasons could be attributed to the lack of widespread success of therapeutic regimens in SUI in the clinical setting. In the case of α-adrenergic medications, the Cochrane review by Alhasso et al. was critical in determining the overall inefficacy of such medications [43]. The abundance of such receptors on numerous organs, especially the cardiovascular system, is a matter of concern. Life-threatening adverse effects such as hemorrhagic stroke and cardiac arrhythmia have limited the success of this drug class [26]. Advances in developing tissue-specific drugs may prove beneficial in the future in diminishing such side effects. Re-evaluating data using novel data synthesis methods, such as the study done by Balk et al. in 2019, may ultimately provide a final answer to whether current available adrenergic medications could be used for SUI. By focusing the endpoints of pharmacotherapy on symptomatic improvement rather than cure, we may observe more favorable outcomes regarding adrenergic medications [12].

It is essential to underscore the significance of neurological pathways in treating SUI. This fact could be reiterated by observing the relative success of duloxetine and other antidepressants [21, 44]. Interestingly, studies on female athletes have shown that those experiencing SUI during practice report fewer incontinence episodes during competition. This could imply the protective effects of neurological mechanisms and heightened catecholamine levels in preserving continence [45]. It would be intriguing to observe whether the recent reassessments of duloxetine, indicating a potential for risks outweighing benefits in the context of stress urinary incontinence (SUI) treatment, might prompt modifications to the existing European guidelines [46]. A thorough review of the pharmacological pathways of the mentioned drugs has been described by Michel et al. [8].

An area of pharmacotherapy that is understudied in SUI patients is the role of novel weight loss medications such as Ozempic and Wegovy (semaglutide). Recent studies have shed light on the correlation between BMI, weight, and waist circumference on the occurrence of SUI [47]. It would be interesting to see if weight loss medications result in any meaningful increase in long-term outcomes.

## Conclusion

The currently available literature on pharmacotherapy options for SUI is rather limited in comparison with novel surgical techniques. The effectiveness and safety of different medication classes for the treatment of SUI have been assessed in numerous trials. The US regulatory authorities have not approved any medication class; whereas some areas such as Europe have approved the use of duloxetine. New target-specific drugs with less systemic side effects may pave the way for the approval of medications. Further research is also necessary to evaluate the role of pharmacotherapy in men in addition to women due to different lower urinary tract characteristics.

## Data Availability

No datasets were generated or analysed during the current study.
